# Genetic insights into number of long-term conditions and their relationship with lifespan

**DOI:** 10.1371/journal.pone.0340181

**Published:** 2026-01-30

**Authors:** Youngjune Bhak, Bruce Guthrie, Albert Tenesa

**Affiliations:** 1 MRC Human Genetics Unit at the MRC Institute of Genetics and Molecular Medicine, University of Edinburgh, Western General Hospital, Edinburgh, United Kingdom; 2 Usher Institute, University of Edinburgh, Edinburgh, United Kingdom; 3 Advanced Care Research Centre, University of Edinburgh, Edinburgh, United Kingdom; 4 The Roslin Institute, University of Edinburgh, Easter Bush Campus, Midlothian, United Kingdom; Curtin University, AUSTRALIA

## Abstract

**Aims:**

Relationships between the genetic risk for long-term conditions (LTCs) and lifespan have been reported. However, the genetic factors underlying the total number of LTCs an individual has (LTC burden) and their association with lifespan have not been fully investigated. This study aims to investigate the genetics of LTC burden and evaluate its relationship with lifespan.

**Methods:**

A genome-wide association study (GWAS) and a genetic heritability test were conducted on LTC burden using data from 343,868 UK Biobank individuals. Global and local genetic correlations between the LTC burden and parental lifespan were estimated. A polygenic risk score (PRS_LTC_) for LTC burden was derived from a separate set of 34,339 UK Biobank individuals with records of age at death, who were not included in the GWAS analysis. The association between the PRS_LTC_ and lifespan, as well as its ability to predict LTC burden, was assessed.

**Results:**

Loci in the *HLA* region were the most significant among the 21 significant independent loci from the GWAS. The estimated SNP heritability of LTC burden was 0.0963 and significantly different from zero (se = 0.0034, p-value = 1.77 x 10^−176^). The global genetic correlation between LTC burden and parental lifespan exhibited a significant global genetic correlation of −0.7869 (se = 0.0419, p-value 9.57 x 10^−79^). Additionally, 44 loci showed significant local genetic correlations (p-value < 2.23 x 10^−5^). Individuals in the highest 10% PRS_LTC_ had, on average, a 0.9-year shorter lifespan and 0.73 more LTCs than those in the lowest 10%.

**Conclusions:**

This study identifies significant genetic factors associated with LTC burden and their association with lifespan, providing insights into the genetic underpinnings of both multiple LTCs and lifespan.

## Introduction

A number of long-term conditions (LTCs), which are chronic conditions requiring lifelong or long-term management, affect human life expectancy [1]. For example, heart failure is associated with a loss of 7.3–20.5 years of life expectancy [2], type 2 diabetes with a loss of 0.99–7.67 years [3], and serious mental illness with a loss of 15–20 years [4]. Multimorbidity, defined as the co-occurrence of multiple LTCs (MLTC), is also associated with a 4.54-5.15-year reduction in life expectancy [5]. The cumulative number of LTCs an individual has, referred to as LTC burden, provides a way to capture overall morbidity load and its potential influence on survival.

There is evidence that genetic factors contribute to both individual LTCs and lifespan. Previous studies have shown that polygenic risk scores (PRSs), which quantify genetic risks for complex diseases and traits, are associated with differences in life expectancy [6], and genome-wide analyses have identified loci associated with human longevity, including the Human Leukocyte Antigen (HLA) region and the Lipoprotein (a) (LPA) gene locus [7,8]. However, most research has focused on single diseases or broad measures of longevity, leaving the genetics of LTC burden and its relationship with lifespan underexplored.

Understanding the shared genetic architecture of LTC burden and lifespan may provide insights into the biological mechanisms that influence both multimorbidity and longevity. Immune dysregulation and chronic inflammation, captured by HLA variation, and lipid metabolism, influenced by LPA, are two plausible biological pathways implicated in these processes [9–11]. Investigating the extent to which LTC burden shares genetic factors with respect to lifespan can help identify potential pathway linking multimorbidity and aging.

In this study, we investigated the genetic basis of LTC burden and its association with lifespan using large-scale UK Biobank data. We performed a genome-wide association study (GWAS) of LTC burden, estimated SNP heritability, and examined both global and local genetic correlations with parental lifespan, which serves as a validated proxy variable for lifespan [12–15]. Finally, we derived a PRS for LTC burden (PRS_LTC_) in 34,339 UK Biobank individuals, who were not included in the GWAS, and evaluated its association with both LTC burden and lifespan to assess the genetic underpinnings of LTC burden and their potential contribution to reduced longevity.

## Materials and methods

### Data source

The UK Biobank (UKB) is a prospective, population-based cohort study that includes comprehensive phenotype and genotype data from approximately 500,000 participants recruited between 2006 and 2010 residing in England, Scotland, and Wales (www.ukbiobank.ac.uk). This open-access resource was established to support investigations into the factors influencing various health outcomes [16]. To ensure homogeneity, we selected unrelated individuals of white ancestry (UK Biobank Data-Field 22006) with less than 10% missing genotypes and with concordance between recorded sex and genetically determined sex. The unrelated participants were identified and extracted using the KING software with the following options: --unrelated --degree2 (version 2.28) [17]. Genotypes of the selected individuals were filtered using PLINK software (version 1.90b) with the following options: --geno 0.01, --hwe 1e-15, --maf 0.01, and mind 0.1, retaining 525,262 variants [18]. Following these selections, the dataset was divided into two sets: 343,868 individuals without a recorded age at death, designated as the training set, and 34,339 individuals with a recorded age at death, designated as the validation set. The training set was used to identify genetic associations and construct polygenic scores for long-term conditions (LTCs), whereas the validation set was used to apply and validate these scores in relation to lifespan.

### Ethics statement

The UK Biobank project was approved by the National Research Ethics Service Committee North West-Haydock (REC reference: 11/NW/0382). Participants provided written informed consent to participate in the UK Biobank. An electronic signed consent was obtained from the participants. This research was conducted using the UK Biobank Resource under projects 44986.

### Study outcomes: LTC classification and characterization

The data including participants’ characteristics and LTCs in the present study were accessible via the UK Biobank Study [16]. The LTC burden in the present study was derived by counting the number of LTCs that each participant had, recorded in hospital inpatient records (UK Biobank Data field 41270). The 32 LTCs examined in this study were defined and categorised based on ICD-10 codes as follows: active asthma (J45 and J46), alcohol problems (E244, F101-F109, G312, G621, G721, I426, K292, K70, K852, K860, Z502, and Z714), anorexia and bulimia (F500-F503), anxiety (F40 and F41), atrial fibrillation (I48), blindness (H540-H542), bronchiectasis (J47 and Q334), cancers (C00-C25, C260, C261, C268, C269, C30-C87, C880, C882, C90-C97, D05, D45, and D46), coronary heart disease (I200, I201, I208, I209, and I21-I25), chronic kidney disease (I120, I131, I132, N183, and N184), chronic obstructive pulmonary disease (J40-J44), chronic liver disease (B150, B160, B190, I85, I982, I983, K70, K700, K704, K711, K72, K743, K754, K758, K760, K762, K763, and K766), dementia (F00, F01, F03, F051, and G30), depression (F32 and F33), diabetes (E10-E14), diverticular disease (K382 and K57), epilepsy (G40 and G41), heart failure (I110, I130, I132, and I50), hypertension (I10-I15), Inflammatory bowel disease (K50, K51, M074, M075, and M076), learning disability (F70-F73, F78, F79, F819, and Q90), multiple sclerosis (G35), psychoactive misuse (F11-F19), Parkinson’s disease (F023 and G20), peripheral vascular disease (I731, I738, I739, I743, I744, and I745), hyperplasia of prostate (N40), rheumatoid arthritis (L405, M02, M03, M070, M071, M072, M073, M08, M09, M315, M316, M353, and M45), schizophrenia and bipolar (F30 and F31), stroke and transient ischemic attack (G450-G454, G458, G459-G468, I61, I630-I635, I638, I639, I64-I66, I691, I693, and I694), thyroid disorders (E035, E038, E039, E050-E052, E055, E058, E059, E062, E063, E065, E069, and H062), dyspepsia (K227, K25-K28), and viral hepatitis (B18) [19].

### Genetic analysis

To investigate genetics of LTC burden, we conducted a GWAS using the training set. For the GWAS, REGENIE software (version 3.2.2) was utilized with default options, except for the following options applied in both step 1 and 2: --apply-rint --qt --maxCatLevels 40 [20]. The block sizes were set to --bsize 1000 for step 1 and --bsize 400 for step 2. Covariates considered in the GWAS include age, age square, sex, age*sex, age square*sex, UK Biobank Centre, UK Biobank genetic array, and the first 20 genetic principal components. To derive independent genetic loci, the GWAS result was clumped to extract index variants using PLINK software with the following options: --clump, --clump-p1 0.00000005, --clump-r2 0.001, and --clump-kb 10000 [18].

To estimate the genetic heritability of LTC burden, we used LD-score regression (version 1.0.1) [21] with default options. LD scores were estimated from the quality-controlled genotype data described above using the following options: --ld-wind-cm 1.0 --l2. An LDSC regression intercept greater than 1.05 was considered indicative of potential residual population stratification, model misspecification, or other artefacts. In our analysis, the intercept was 0.89, indicating no evidence of inflation. The genetic heritability was derived using default options. To investigate the genetic correlation between LTC burden and lifespan, we used GWAS results on parental lifespan from large biobank studies, taking it as a surrogate variable for lifespan [12]. Global and local genetic correlations between were estimated using High-Definition Likelihood (HDL, version 1.4.0) and LAVA software (version 0.1.0), respectively with default option [22,23]. LD references to use in HDL were generated from the quality-controlled genotype data using build_ld_ref functions of HDL software with default options. For LAVA, we used LD reference data (g1000_eur) and locus definition file (blocks_s2500_m25_f1_w200.GRCh37_hg19.locfile covering 2,495 regions) provided by LAVA. Only 2,247 of the 2,496 regions were able to be analysed and a Bonferroni-corrected significance threshold of p < 0.05/2,247 (2.23 x 10^−10^) was applied to account for the number of tests accordingly. To investigate the genetic risk of LTC burden in relation to lifespan, we derived a PRS for LTC burden in the validation set by using the GWAS result on LTC burden in the training set using PRSice-2 software (version 2.3.3) with options specifying column names and --binary-target F [24].

### Statistical analyses

Categorical variables were presented as counts and percentages, and continuous variables were presented as means and standard deviations (SD). The correlations between the PRS and both LTC burden and lifespan were assessed using Spearman’s rank-order correlation. LTC burden and lifespan between the PRS groups were compared using Wilcoxon signed-rank test. To account for the multiple tests, we applied thresholds for significance based on Bonferroni correction. All statistical analyses not performed using the software described were conducted in R (version 4.4.3).

## Results

A total of 343,868 individuals who were alive at the end of follow-up comprised the training set, and 34,339 individuals known to have died, with records of age at death, comprised the validation set (S1 Table). At recruitment to the UK Biobank study, the average age in the training set was 56.5 years (SD 7.9) compared with 61.9 years (SD 6.3) in the validation set. The training set had a lower proportion of males than the validation set (44.9% vs 59.7%). Similarly, the average number of LTCs was 1.41 (SD 1.77) in the training and 3.54 (SD 2.44) in the validation set. The average age at death in the validation set was 71.2 years (SD 7.5), with males averaging 71.0 years (SD = 7.63) and females 71.3 years (SD = 7.33). The difference between sexes was statistically significant (p-value = 5.0 x 10^−4^).

**Fig 1:**
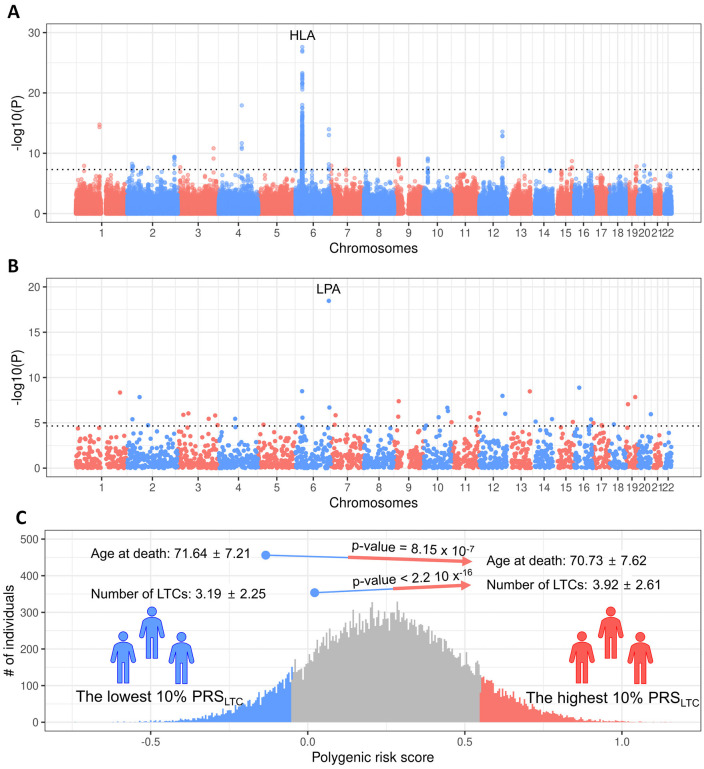
Genetics of LTC burden and its association with lifespan. Panel A shows a Manhattan plot of GWAS results for LTC burden in the training set (n = 343,868). Each point represents a genetic variant plotted by chromosomal position (x-axis) and –log₁₀ p-value (y-axis). The dashed horizontal line marks the genome-wide significance threshold (p = 5 x 10^−8^). Panel B shows a Manhattan plot of local genetic correlations between LTC burden and parental lifespan, with each point representing a genomic region. The dashed line marks the Bonferroni-corrected significance threshold (p = 2.23 x 10^−5^). The most significant regions include the HLA (Panel A) and *LPA* (Panel B) loci. Panel C shows the distribution of the polygenic risk score for LTC burden (PRS_LTC) in the validation set (n = 34,339). The lowest 10% (blue) and highest 10% (red) PRS_LTC groups are compared for age at death and number of LTCs, with mean ± SD values and p-values shown for group differences.

To investigate the genetics of LTC burden, we conducted a GWAS using the training set. After LD-based clumping, 21 independent genetic loci were identified (Fig 1A, S2 Table). The most significant association, rs532965, was observed in the intron of human leukocyte antigen class II gene *HLA-DRB1* (S3 Table). The SNP heritability of LTC burden was modest but statistically significant, estimated at 0.0963 (se = 0.0034, p-value = 1.77 x 10^-176^, regression intercept = 0.887 (0.101)).

To assess global and local genetic correlation between LTC burden and lifespan, we used a previously published GWAS result of parental lifespan as a surrogate for lifespan. The global genetic correlation between LTC burden and lifespan was −0.7869 (se = 0.0419, p-value 9.57 x 10^−79^). There were 44 significant negative local genetic correlations between LTC burden and lifespan out of the 2,247 loci assessed (p-value < 2.23 x 10^−5^, Fig 1B, S4 Table). The most significant negative local correlation encompassed the lipoprotein (a) (LPA) gene region (chr6: 160,583,919–161,371,014).

To examine the association between genetic risk for the LTC burden and lifespan, we derived a PRS_LTC_ in the validation set based on the GWAS result for LTC burden from the training set. The PRS_LTC_ was positively correlated with LTC burden (Spearman’s coefficient = 0.081, p-value < 2.2 x 10^−16^) and negatively correlated with age at death (Spearman’s coefficient = −0.032, p-value = 2.53 x 10^−9^). Individuals in the highest 10% of the PRS_LTC_ had, on average, 0.73 more LTCs at the time of recruitment (p-value < 2.2 x 10^−16^) and a 0.9-year shorter lifespan (p-value = 8.15 x 10^−7^) compared to individuals with the lowest 10% of the PRS_LTC_ (Fig 1C). Although statistically significant, these differences were modest in magnitude.

In sex-stratified analyses, males in the highest PRS_LTC_ had a shorter average lifespan (70.80 years, SD = 7.53) compared with those in the lowest decile (71.92 years, SD = 7.04; p-value = 2.13 x 10^-6^), while females showed a similar but weaker and not significant difference (70.62 years, SD = 7.77 vs. 71.28 years, SD = 7.46; p-value = 0.041). The LTC burden increased with higher PRS_LTC_ in both sexes, rising from 3.31 (SD = 2.29) to 4.09 (SD = 2.62) in males (p-value < 2.2 x 10^-16^) and from 3.01 (SD = 2.18) to 3.67 (SD = 2.54) in females (p-value = 1.10 x 10^-11^).

## Discussion

The most significant loci identified in the GWAS conducted in this study were located in the HLA region, which is known to play an important role in immunity and inflammation and has been linked to various long-term conditions [25–28]. Given the previously reported associations between immunity, inflammation, and MLTC, our findings provide genetic-level insights into the relationship between immune function and LTC burden [9–11,29]. The local genetic correlation analysis also revealed the most significant signal in the LPA gene region. The LPA gene has been reported to have genetic associations with coronary artery disease, diabetes, and depression, and has also been shown to have both phenotypic and genetic links to lifespan [7,30–33]. The local genetic correlation observed in the LPA gene region between LTC burden and lifespan in this study may offer genetic insights into the lethal, lifespan-shortening components of MLTC.

The associations observed at the *HLA* and *LPA* loci may indicate that both inflammation and lipid metabolism contribute to ageing and LTC burden. Variants in *HLA* can influence immune activation and chronic inflammation, while *LPA* variants regulate lipoprotein(a) levels, a known risk factor for atherosclerosis and vascular disease [7]. Previous studies suggest that these mechanisms may act through independent pathways but could also interact through shared mechanisms such as endothelial dysfunction and oxidative stress [7,34,35]. Although our study was not designed to test this directly, these findings highlight the potential link between immune and lipid pathways in the development of age-related multimorbidity. Future studies should investigate whether *HLA* and *LPA* act synergistically or independently to influence disease progression, aging, and lifespan.

The observed associations between PRS_LTC_, LTC burden, and lifespan suggest a shared genetic architecture between the accumulation of LTCs and reduced longevity. However, the magnitude of these associations was modest. This may indicate that while genetic factors contribute to both LTC burden and lifespan, environmental and lifestyle factors are also likely to play a substantial role.

This study provides insights into the genetics of LTC burden and its association with lifespan using a large-scale dataset with systematic ascertainment of long-term conditions. However, several limitations warrant cautious interpretation. First, there are various ways to define each LTC, and this study only included a subset of common LTCs based on hospital inpatient records. This reliance on hospital-derived phenotypes may introduce misclassification, incomplete case capture, and censoring, potentially affecting the accuracy of the LTC burden estimates. Second, the interplay between different LTCs, lifestyle, and environmental factors—each of which may amplify or mitigate disease impact relative to a simple summation of conditions—was not examined in the present study. Third, analyses were limited to individuals of European ancestry, restricting generalisability and underscoring the need for replication in diverse populations. Fourth, the genetic correlation analyses should be interpreted as reflecting shared genetic signal rather than causality, and their accuracy may be affected by factors such as LD reference panels, block definitions, SNP density, and local heritability estimates. Fifth, although comprehensive genotype QC and imputation procedures were applied, inaccuracies may persist—particularly in regions with low SNP density—which could influence downstream analyses. In addition, while the PRS analyses provided meaningful associations, the predictive performance of PRS for complex traits such as LTC burden is generally modest and should be interpreted accordingly. Finally, LTC burden as defined here aggregates heterogeneous conditions, ranging from relatively mild and common to severe and life-limiting, meaning that the observed genetic signals may reflect shared biological pathways as well as strong effects attributable to specific high-impact LTCs. Future studies should evaluate stratified or severity-weighted LTC burdens, incorporate environmental and sociodemographic factors, and use multi-ethnic datasets to more comprehensively characterise the genetic and non-genetic determinants of multimorbidity.

## Supporting information

S1 Table Baseline characteristics of the study population. (XLSX)

S2 Table Independent genome-wide significant loci from the GWAS on LTC burden. (XLSX)

S3 Table Annotations of associated variants. (XLSX)

S4 Table Local genetic correlations between the LTC burdens and parental lifespan. (XLSX)

## References

[1] Naghavi M, Ong KL, Aali A, Ababneh HS, Abate YH, Abbafati C, et al. Global burden of 288 causes of death and life expectancy decomposition in 204 countries and territories and 811 subnational locations, 1990–2021: a systematic analysis for the Global Burden of Disease Study 2021. The Lancet. 2024;403(10440):2100–32. 10.1016/S0140-6736(24)00367-2 PMC11126520 38582094

[2] Hariharaputhiran S, Peng Y, Ngo L, Ali A, Hossain S, Visvanathan R, et al. Long-term survival and life expectancy following an acute heart failure hospitalization in Australia and New Zealand. Eur J Heart Fail. 2022;24(9):1519–28. doi: 10.1002/ejhf.2595 35748124 PMC9804480

[3] Wang B, Fu Y, Tan X, Wang N, Qi L, Lu Y. Assessing the impact of type 2 diabetes on mortality and life expectancy according to the number of risk factor targets achieved: an observational study. BMC Med. 2024;22(1):114. doi: 10.1186/s12916-024-03343-w 38475845 PMC10935790

[4] Nordentoft M, Wahlbeck K, Hällgren J, Westman J, Osby U, Alinaghizadeh H, et al. Excess mortality, causes of death and life expectancy in 270,770 patients with recent onset of mental disorders in Denmark, Finland and Sweden. PLoS One. 2013;8(1):e55176. doi: 10.1371/journal.pone.0055176 23372832 PMC3555866

[5] Chudasama YV, Khunti KK, Zaccardi F, Rowlands AV, Yates T, Gillies CL, et al. Physical activity, multimorbidity, and life expectancy: a UK Biobank longitudinal study. BMC Med. 2019;17(1):108. doi: 10.1186/s12916-019-1339-0 31186007 PMC6560907

[6] Hu D, Li Y, Zhang D, Ding J, Song Z, Min J, et al. Genetic trade-offs between complex diseases and longevity. Aging Cell. 2022;21(7):e13654. doi: 10.1111/acel.13654 35754110 PMC9282840

[7] Joshi PK, Pirastu N, Kentistou KA, Fischer K, Hofer E, Schraut KE, et al. Genome-wide meta-analysis associates HLA-DQA1/DRB1 and LPA and lifestyle factors with human longevity. Nat Commun. 2017;8(1):910. doi: 10.1038/s41467-017-00934-5 29030599 PMC5715013

[8] Zenin A, Tsepilov Y, Sharapov S, Getmantsev E, Menshikov LI, Fedichev PO, et al. Identification of 12 genetic loci associated with human healthspan. Commun Biol. 2019;2:41. doi: 10.1038/s42003-019-0290-0 30729179 PMC6353874

[9] Friedman E, Shorey C. Inflammation in multimorbidity and disability: An integrative review. Health Psychol. 2019;38(9):791–801. doi: 10.1037/hea0000749 31436464 PMC6709716

[10] Han S, Li S, Yang Y, Liu L, Ma L, Leng Z, et al. Mapping multimorbidity progression among 190 diseases. Commun Med (Lond). 2024;4(1):139. doi: 10.1038/s43856-024-00563-2 38992158 PMC11239867

[11] Nakayama Y, Fujiu K, Oshima T, Matsuda J, Sugita J, Matsubara TJ, et al. Heart failure promotes multimorbidity through innate immune memory. Sci Immunol. 2024;9(95):eade3814. doi: 10.1126/sciimmunol.ade3814 38787963

[12] Timmers PR, Mounier N, Lall K, Fischer K, Ning Z, Feng X, et al. Genomics of 1 million parent lifespans implicates novel pathways and common diseases and distinguishes survival chances. Elife. 2019;8:e39856. doi: 10.7554/eLife.39856 30642433 PMC6333444

[13] Vilar-Ribó L, Cabana-Domínguez J, Martorell L, Ramos-Quiroga JA, Sanchez-Roige S, Palmer AA, et al. Shared genetic architecture between attention-deficit/hyperactivity disorder and lifespan. Neuropsychopharmacology. 2023;48(7):981–90. doi: 10.1038/s41386-023-01555-x 36906694 PMC10209393

[14] Ye C-J, Kong L-J, Wang Y-Y, Dou C, Zheng J, Xu M, et al. Mendelian randomization evidence for the causal effects of socio-economic inequality on human longevity among Europeans. Nat Hum Behav. 2023;7(8):1357–70. doi: 10.1038/s41562-023-01646-1 37386110

[15] Ying K, Zhai R, Pyrkov TV, Shindyapina AV, Mariotti M, Fedichev PO, et al. Genetic and phenotypic analysis of the causal relationship between aging and COVID-19. Commun Med (Lond). 2021;1:35. doi: 10.1038/s43856-021-00033-z 35602207 PMC9053191

[16] Sudlow C, Gallacher J, Allen N, Beral V, Burton P, Danesh J, et al. UK biobank: an open access resource for identifying the causes of a wide range of complex diseases of middle and old age. PLoS Med. 2015;12(3):e1001779. doi: 10.1371/journal.pmed.1001779 25826379 PMC4380465

[17] Manichaikul A, Mychaleckyj JC, Rich SS, Daly K, Sale M, Chen W-M. Robust relationship inference in genome-wide association studies. Bioinformatics. 2010;26(22):2867–73. doi: 10.1093/bioinformatics/btq559 20926424 PMC3025716

[18] Purcell S, Neale B, Todd-Brown K, Thomas L, Ferreira MAR, Bender D, et al. PLINK: a tool set for whole-genome association and population-based linkage analyses. Am J Hum Genet. 2007;81(3):559–75. doi: 10.1086/519795 17701901 PMC1950838

[19] Barnett K, Mercer SW, Norbury M, Watt G, Wyke S, Guthrie B. Epidemiology of multimorbidity and implications for health care, research, and medical education: a cross-sectional study. Lancet. 2012;380(9836):37–43. doi: 10.1016/S0140-6736(12)60240-2 22579043

[20] Mbatchou J, Barnard L, Backman J, Marcketta A, Kosmicki JA, Ziyatdinov A, et al. Computationally efficient whole-genome regression for quantitative and binary traits. Nat Genet. 2021;53(7):1097–103. doi: 10.1038/s41588-021-00870-7 34017140

[21] Bulik-Sullivan BK, Loh P-R, Finucane HK, Ripke S, Yang J, Schizophrenia Working Group of the Psychiatric Genomics Consortium, et al. LD Score regression distinguishes confounding from polygenicity in genome-wide association studies. Nat Genet. 2015;47(3):291–5. doi: 10.1038/ng.3211 25642630 PMC4495769

[22] Ning Z, Pawitan Y, Shen X. High-definition likelihood inference of genetic correlations across human complex traits. Nat Genet. 2020;52(8):859–64. doi: 10.1038/s41588-020-0653-y 32601477

[23] Werme J, van der Sluis S, Posthuma D, de Leeuw CA. An integrated framework for local genetic correlation analysis. Nat Genet. 2022;54(3):274–82. doi: 10.1038/s41588-022-01017-y 35288712

[24] Choi SW, O’Reilly PF. PRSice-2: Polygenic Risk Score software for biobank-scale data. Gigascience. 2019;8(7):giz082. doi: 10.1093/gigascience/giz082 31307061 PMC6629542

[25] Canela-Xandri O, Rawlik K, Tenesa A. An atlas of genetic associations in UK Biobank. Nat Genet. 2018;50(11):1593–9. doi: 10.1038/s41588-018-0248-z 30349118 PMC6707814

[26] Ricaño-Ponce I, Wijmenga C. Mapping of immune-mediated disease genes. Ann Rev Genomics Human Genet. 2013;14(1):325–53. 23834318 10.1146/annurev-genom-091212-153450

[27] Horton R, Wilming L, Rand V, Lovering RC, Bruford EA, Khodiyar VK, et al. Gene map of the extended human MHC. Nat Rev Genet. 2004;5(12):889–99. doi: 10.1038/nrg1489 15573121

[28] Kumar V, Wijmenga C, Xavier RJ. Genetics of immune-mediated disorders: from genome-wide association to molecular mechanism. Curr Opin Immunol. 2014;31:51–7. doi: 10.1016/j.coi.2014.09.007 25458995 PMC5080657

[29] Barnes PJ. Mechanisms of development of multimorbidity in the elderly. Eur Respir J. 2015;45(3):790–806. doi: 10.1183/09031936.00229714 25614163

[30] Nelson CP, Goel A, Butterworth AS, Kanoni S, Webb TR, Marouli E, et al. Association analyses based on false discovery rate implicate new loci for coronary artery disease. Nat Genet. 2017;49(9):1385–91. doi: 10.1038/ng.3913 28714975

[31] Baltramonaityte V, Pingault J-B, Cecil CAM, Choudhary P, Järvelin M-R, Penninx BWJH, et al. A multivariate genome-wide association study of psycho-cardiometabolic multimorbidity. PLoS Genet. 2023;19(6):e1010508. doi: 10.1371/journal.pgen.1010508 37390107 PMC10343069

[32] Arsenault BJ, Pelletier W, Kaiser Y, Perrot N, Couture C, Khaw K-T, et al. Association of Long-term Exposure to Elevated Lipoprotein(a) Levels With Parental Life Span, Chronic Disease-Free Survival, and Mortality Risk: A Mendelian Randomization Analysis. JAMA Netw Open. 2020;3(2):e200129. doi: 10.1001/jamanetworkopen.2020.0129 32108890 PMC7049087

[33] Berg K, Rø O, editors. Lp (a) lipoprotein level and longevity. Annales de Genetique. 1991. 1839706

[34] Dahlén GH, Slunga L, Lindblom B. Importance of Lp(a) lipoprotein and HLA genotypes in atherosclerosis and diabetes. Clin Genet. 1994;46(1 Spec No):46–51. doi: 10.1111/j.1399-0004.1994.tb04201.x 7988077

[35] Missala I, Kassner U, Steinhagen-Thiessen E. A Systematic Literature Review of the Association of Lipoprotein(a) and Autoimmune Diseases and Atherosclerosis. Int J Rheumatol. 2012;2012:480784. doi: 10.1155/2012/480784 23304154 PMC3523136

