# Supplementary material for: Genetic insights into number of long-term conditions and their relationship with lifespan
Source: PLoS One. 2026 Jan 30;21(1):e0340181. doi: 10.1371/journal.pone.0340181 (PMC12857943; doi:10.1371/journal.pone.0340181)
Supplement: Attachment — Submitted filename: PONE-D25-09280 TO THE EDITOR.pdf [file pone.0340181.s006.pdf]

University of Baghdad/
College of Science for Women
Biology department
Baghdad
11/7 /2025

Dear Editors in PLOS ONE,

I hope you are fine.

Thank you to give me the opportunity to review the manuscript PONE-D-25-09280 entitled (Genetic insights into number of long-term conditions and their relationship with lifespan.

The manuscript sheds light on the polygenetic causes that may be related to reduced lifespan in the white ancestry population in the UK by analysis of data from the UK Biobank.

It's noteworthy the large number of data included in the study (343868 alive individuals and 34339 dead individuals).

Some clarity is needed in the following points:

-The term (the long-term condition) is not defined; it includes the risk of diseases that reduce lifespan, the environment and socioeconomic conditions, and the quality of life during the last years before death.

- Why the study depends on the genetic correlation with parents' lifespan rather than the data of the same individuals in the study.

- Please specify the studied genetic loci in a table with their importance or correlation with studied LTC.

- The categorization of the 32 LTC in the lines (13-31) is not clear.

- The results of the studied data do not explain if sex has any correlation with lifespan.

- The study does not specify which type/s or gene/s of HLA and LPA are the most frequently associated with aging or reduced lifespan.

The manuscripts need a revision.

Best regards,

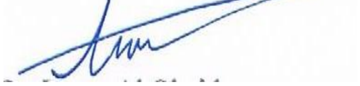

Assist. Prof. Luma Hassan Alwan Al Obaidy
11/ 7 / 2025
