# Supplementary material for: Genetic insights into number of long-term conditions and their relationship with lifespan
Source: PLoS One. 2026 Jan 30;21(1):e0340181. doi: 10.1371/journal.pone.0340181 (PMC12857943; doi:10.1371/journal.pone.0340181)
Supplement: Attachment — Submitted filename: Manuscript Review for PONE-D-25-09280.docx [file pone.0340181.s005.docx]

**Manuscript Review for PLOS ONE**

**Manuscript ID:** PONE-D-25-09280

**Title:** Genetic insights into number of long-term conditions and their relationship with lifespan.

**Corresponding author:** Youngjune Bhak Ph.D., Albert Tenesa Ph.D.

**Overall assessment:**

While this manuscript presents valuable clinical insights, it falls short of meeting the genetic scope expected for a study of this nature. The authors primarily approach the research question from a medical perspective, with limited emphasis on genetic mechanisms, analysis depth, and biological interpretation. Given the study’s aim to investigate genetic associations, the lack of molecular genetic discussion, pathway-based integration, and hypothesis-driven synthesis significantly weakens its contribution to the field. The study fails to articulate how these genetic discoveries could inform future research (e.g., functional studies, multi-omics integration) or clinical applications (e.g., personalized risk stratification, therapeutic targeting).

Due to these critical limitations, I **do not recommend acceptance** of the manuscript in its current form. Substantial revisions—particularly in expanding the genetic analysis, refining the discussion, and strengthening the biological rationale—are required before reconsideration for publication.

**Major Comments**

**1. Introduction**

**- Background Depth**: The introduction should provide a more thorough review of existing literature on genetic factors relevant to the study. Expanding on key genetic mechanisms, prior evidence, and unresolved questions would better contextualize the study’s significance.

**- Final Paragraph Revision**: The concluding paragraph currently summarizes methodological aspects rather than clearly stating the study’s aims, hypothesis, and research questions.

**2. Methods**

**- Ethics Statement Placement**: To improve logical flow, move the ethics approval statement to the *Data Sources* subsection, where ethical considerations related to data collection are typically addressed.

**- Subheading Accuracy**: The *Study Outcome* subheading is misleading, as the content describes long-term condition (LTC) categorizations. Rename this subsection (e.g., *LTC Classification and Characterization*) to accurately reflect its content.

**- Group Nomenclature**: Clarify the rationale for labeling cohorts as *Application Group* and *Discovery Group*. Justify these terms in the context of the study design.

**3. Discussion**

**Lack of Integration Between Key Findings**
The discussion currently presents each result in isolation without synthesizing them into a cohesive narrative. For instance, the GWAS identified HLA as the most significant locus (linked to immunity/inflammation), while the local genetic correlation analysis highlighted LPA as the top candidate. However, the author fails to explore potential biological or mechanistic connections between these findings. A more impactful discussion would:

- Hypothesize how inflammation-related pathways (via HLA) might interact with LPA-mediated lipid metabolism to influence the observed phenotype.
- Address whether these loci operate independently or synergistically in disease pathogenesis, citing prior evidence (e.g., shared pathways like endothelial dysfunction or oxidative stress).
- Propose a research gap: "Given the dual prominence of immune and lipid-related genes, future studies should investigate crosstalk between these pathways in [disease context]."
