# Supplementary material for: Genetic insights into number of long-term conditions and their relationship with lifespan
Source: PLoS One. 2026 Jan 30;21(1):e0340181. doi: 10.1371/journal.pone.0340181 (PMC12857943; doi:10.1371/journal.pone.0340181)
Supplement: Attachment — Submitted filename: Response to reviewers.docx [file pone.0340181.s007.docx]

**ACADEMIC EDITOR:**

We would like to sincerely thank the Academic Editor and reviewers for their thorough evaluation of our manuscript and for providing constructive and insightful comments. We have carefully considered all suggestions and have revised the manuscript accordingly. These revisions have improved the clarity, presentation, and interpretability of our work. Below, we provide a detailed, point-by-point response to each comment, indicating the changes made and where they can be found in the revised manuscript.

1. Ensure the manuscript follows the journal’s style and formatting requirements.

- Thank you for your guidance. We have carefully reviewed the PLOS ONE submission guidelines and revised the manuscript accordingly. Tables, figures, references, and line spacing now follow the journal’s style requirements.

2. Moderate language around causality. For example, instead of using terms like ‘impact,’ consider using ‘association’ where appropriate.

- We have revised the manuscript to avoid causal language. The term ‘impact’ in the Abstract section has been replaced with ‘association’.

3. Better account for heterogeneity in the LTC burden definition. For example, different conditions may vary in severity and genetic basis, which could affect interpretation.

- We have added text in the Discussion sections to acknowledge and account for heterogeneity in LTC burden. Specifically, we note that LTC burden combines conditions with differing severity, chronicity, prevalence, and genetic architecture. This heterogeneity may influence both the genetic signals observed and their interpretation. We have discussed this as a limitation and suggested future analyses stratifying LTCs by severity, mortality risk, or genetic heritability to refine interpretation.

4. Clarify results presentation and expand the interpretive discussion. For example, include more detail on PRS performance and improve figure legends for clarity.

- We thank the reviewer for this valuable suggestion. In the Results section, we have now noted the direction and modest magnitude of correlation between PRS for LTC, LTC burden, and age at death. In the Discussion section, we have expanded our interpretation of the PRS findings, emphasising the modest effect sizes observed, and the likely contributions of environmental and lifestyle factors. We have also revised the **Figure 1 legend** to provide clearer descriptions of each panel, the statistical thresholds used, and the group comparisons shown, so that the figure can be interpreted without referring to the main text. These changes improve the clarity and interpretability of our results without altering the underlying analyses.

5. Correct minor typographical and grammatical errors throughout the manuscript.

- We have performed a thorough proofread of the manuscript to correct typographical and grammatical errors, improve sentence clarity, and ensure consistency in terminology (e.g., “UK BioBank” corrected to “UK Biobank,” “less males” changed to “lower proportion of males”).

6. Provide information availability of GWAS summary data from their work.

- We have added a Data Availability Statement specifying that the GWAS summary statistics generated in this study will be made publicly available upon the publish.

**Journal requirements:**

When submitting your revision, we need you to address these additional requirements.

1. Please ensure that your manuscript meets PLOS ONE's style requirements, including those for file naming. The PLOS ONE style templates can be found at

<https://journals.plos.org/plosone/s/file?id=wjVg/PLOSOne_formatting_sample_main_body.pdf> and

<https://journals.plos.org/plosone/s/file?id=ba62/PLOSOne_formatting_sample_title_authors_affiliations.pdf>

🡪 We have revised the manuscript to meet PLOS ONE formatting guidelines, including file naming, structure, and layout, in accordance with the provided templates.

2. When completing the data availability statement of the submission form, you indicated that you will make your data available on acceptance. We strongly recommend all authors decide on a data sharing plan before acceptance, as the process can be lengthy and hold up publication timelines. Please note that, though access restrictions are acceptable now, your entire data will need to be made freely accessible if your manuscript is accepted for publication. This policy applies to all data except where public deposition would breach compliance with the protocol approved by your research ethics board. If you are unable to adhere to our open data policy, please kindly revise your statement to explain your reasoning and we will seek the editor's input on an exemption. Please be assured that, once you have provided your new statement, the assessment of your exemption will not hold up the peer review process.

🡪 We confirm that all data underlying the findings are now publicly available. The genome-wide association study (GWAS) summary statistics have been deposited in Zenodo under the DOI 10.5281/zenodo.17272288

. Data from the UK Biobank are accessible to qualified researchers upon application and approval (https://www.ukbiobank.ac.uk/enable-your-research/apply-for-access). The revised Data Availability statement reflects these updates and complies with PLOS ONE’s open data policy (page 13, lines 10 to 16).

3. Please amend either the abstract on the online submission form (via Edit Submission) or the abstract in the manuscript so that they are identical.

🡪 We have reviewed and amended the abstract to ensure that the version in the online submission form is identical to the abstract in the manuscript.

**Reviewers**

Reviewer #1: This study addresses an important and timely question: the genetic underpinnings of long-term condition (LTC) burden and its relationship with lifespan. By leveraging the large-scale UK Biobank resource, the authors provide valuable insights into shared genetic architecture between LTC burden and parental lifespan, with potential implications for understanding biological aging and healthspan. The study is well-conceived, but several aspects of the manuscript require clarification and improvement to ensure transparency, accuracy, and interpretability. I recommend major revision.

“At recruitment to the UK Biobank study, average age in the Discovery group was 56.5 (SD 7.9) years vs 61.9 (SD 6.3) years in the Application group, the Discovery group had less males than the Application group (44.9% vs 59.7%)” should be revised to:
“At recruitment to the UK Biobank study, average age in the Discovery group was 56.5 (SD 7.9) years vs 61.9 (SD 6.3) years in the Application group, and the Discovery group had fewer males than the Application group (44.9% vs 59.7%).”

“Similarly, the average number of LTCs was 1.41 (SD 1.77) lower in the Discovery than the Application group (3.54, SD 2.44)” should be revised to:
“Similarly, the average number of LTCs was lower in the Discovery (1.41, SD 1.77) than the Application group (3.54, SD 2.44).”
Otherwise, it may be interpreted as “1.41 times lower.”

🡪 We sincerely thank the reviewer for this helpful suggestion. We have revised the sentences to improve clarity and to avoid any potential ambiguity in interpretation (Page 5, lines 9 to 18).

I suggest editing Supplementary Table 2 to include the gene or genomic location of the significantly associated SNPs. If these SNPs are intergenic, that should be explicitly stated. I also suggest including a brief mention of these significant associations in the main Results section.
🡪 We thank the reviewer for this helpful suggestion. We have added variant annotations, including gene or genomic location, in Supplementary Table 3. For variants in the HLA region, rs532965, we have explicitly stated the HLA subtype and the variant’s location within the gene. In addition, we have briefly mentioned these significant associations in the main Results section (Page 5, lines 21 to 24).

Please clarify or provide evidence supporting the use of parental lifespan as a valid surrogate for lifespan. A brief justification or citation would be appropriate.

🡪 We thank the reviewer for raising this important point. We have added a justification in the Methods section, citing previous large-scale GWAS (e.g., Timmers et al. *eLife* 2019; Joshi et al. *Nat Commun* 2017) that used parental lifespan as a validated surrogate for individual lifespan (page 9, lines 9 to 13).

Consider revising the visual in Panel C. The “little people” icon showing individuals with canes to represent the top 10% PRS group (who actually died younger) could be misleading.
🡪 We thank the reviewer for this valuable feedback. To avoid any potential misinterpretation, we have revised Panel C to use a unified icon for all groups.

I strongly recommend stratifying analyses by sex and reporting findings accordingly.

🡪 We thank the reviewer for this valuable suggestion. We have now performed sex-stratified analyses and incorporated the results into the *Results* section. Specifically, males in the highest PRS_LTC_ decile had a shorter average lifespan (70.80 years, SD = 7.53) compared with those in the lowest decile (71.92 years, SD = 7.04; p = 2.13E-06), while females showed a weaker and not significant difference (70.62 years, SD = 7.77 vs. 71.28 years, SD = 7.46; p = 0.041). The LTC burden also increased with higher PRS_LTC_ in both sexes. These findings are now described in the Results section (page 7, lines 16 to 21).

The reporting of findings could be improved. For reference, see: PMID 33692554.

🡪 We thank the reviewer for this helpful suggestion. We have thoroughly revised the manuscript to improve the reporting of findings, taking into account the recommended reference.

In the Discussion, line 32, "LTC" should be used in place of the incorrect term.

🡪 We thank the reviewer for noticing this error. We have corrected ‘LCT’ to ‘LTC’ in the Discussion.

The Discussion overall is too brief and superficial. I encourage the authors to expand it to better contextualize the findings and acknowledge limitations.
🡪 We thank the reviewer for this valuable suggestion. We have substantially expanded the Discussion to provide greater context for our findings and to more fully acknowledge the study’s limitations.

Please provide a brief description of the parental lifespan GWAS used in the Methods section to aid readers in understanding the study design.

🡪 We thank the reviewer for this helpful comment. In the Introduction and Methods section, we have added a brief description of the parental lifespan GWAS, noting that we used summary statistics from large biobank studies (page 5, lines 2 to 3 and page 12, lines 9 to 12). This addition clarifies the data source and addresses the reviewer’s concern.

The authors do not explicitly state whether all data underlying their findings have been made available. However, the primary dataset used, the UK Biobank, is accessible to qualified researchers upon application and approval from the UK Biobank. I recommend that the authors clarify this in the manuscript. Additionally, the authors do not mention whether their GWAS summary statistics will be made available. Please include this information.

🡪 We thank the reviewer for this helpful suggestion. We have revised the *Data Availability* section to clarify data access procedures. Specifically, we now state that the data used in this study are part of the UK Biobank resource, which is accessible to qualified researchers upon application and approval. In addition, we have made the GWAS summary statistics generated and analysed in this study publicly available on Zenodo (DOI: 10.5281/zenodo.17272288), which can be accessed without restriction for reuse and further analysis (Page 13, lines 10 to 16).

Reviewer #2: The study conducted by the authors are too predictive and ignored the real time conditions such as lifestyle of individuals which largely affect these long term conditions and life expectancy. Though the number of subjects are good enough to predict a strong conclusion but the outcome of study (HLA region, LPA & higher PRS value for shorter life span) is already known and described in different published studies as authors also cited.

🡪 We thank the reviewer for these thoughtful comments. We agree that lifestyle and environmental factors play an important role in long-term conditions and lifespan. While these factors were beyond the scope of our genetic analysis, we now acknowledge this limitation more explicitly in the Discussion (From page 8, line 23 to page 9, line 17). We also note that, although associations in the *HLA* region, *LPA*, and polygenic risk scores for lifespan have been previously reported, our study extends this literature by focusing on LTC burden as an outcome. We believe this adds value by reinforcing and contextualizing prior findings in a new framework.

Reviewer #3: Manuscript Review for PLOS ONE
Manuscript ID: PONE-D-25-09280
Title: Genetic insights into number of long-term conditions and their relationship with lifespan.
Corresponding author: Youngjune Bhak Ph.D., Albert Tenesa Ph.D.

Overall assessment:
While this manuscript presents valuable clinical insights, it falls short of meeting the genetic scope expected for a study of this nature. The authors primarily approach the research question from a medical perspective, with limited emphasis on genetic mechanisms, analysis depth, and biological interpretation. Given the study’s aim to investigate genetic associations, the lack of molecular genetic discussion, pathway-based integration, and hypothesis-driven synthesis significantly weakens its contribution to the field. The study fails to articulate how these genetic discoveries could inform future research (e.g., functional studies, multi-omics integration) or clinical applications (e.g., personalized risk stratification, therapeutic targeting).

Major Comments
1. Introduction
- Background Depth: The introduction should provide a more thorough review of existing literature on genetic factors relevant to the study. Expanding on key genetic mechanisms, prior evidence, and unresolved questions would better contextualize the study’s significance.

🡪 We agree and have substantially expanded the Introduction. We now review previous genome-wide studies of longevity, highlighting that genetic loci such as HLA, and LPA have been robustly associated with lifespan in large-scale GWAS. We also discuss biological pathways—immune dysregulation and chronic inflammation (HLA), and lipid metabolism (LPA)—that may plausibly link multimorbidity and reduced lifespan. These additions place our study within the broader context of known genetic contributors to longevity (page 4, lines 10 to 22).

- Final Paragraph Revision: The concluding paragraph currently summarizes methodological aspects rather than clearly stating the study’s aims, hypothesis, and research questions.

🡪 We thank the reviewer for this constructive comment. We have revised the final paragraph of the *Introduction* to clearly state the study’s aims and design (From age 4, line 23 to page 5, line 6). Specifically, the revised paragraph now outlines our objectives: (1) to conduct a GWAS of long-term condition (LTC) burden and estimate SNP heritability, (2) to assess both global and local genetic correlations with parental lifespan as a proxy for longevity, and (3) to evaluate the association between a polygenic risk score for LTC burden (PRS_LTC) and both LTC burden and lifespan in an independent group.

2. Methods
- Ethics Statement Placement: To improve logical flow, move the ethics approval statement to the Data Sources subsection, where ethical considerations related to data collection are typically addressed.

🡪 We thank the reviewer for this suggestion. We have moved the ethics approval statement to the *Data Sources* subsection in the revised manuscript to improve clarity and logical flow.

- Subheading Accuracy: The Study Outcome subheading is misleading, as the content describes long-term condition (LTC) categorizations. Rename this subsection (e.g., LTC Classification and Characterization) to accurately reflect its content.

🡪 We appreciate the reviewer’s helpful observation. We have revised the subheading *Study Outcome* to *LTC Classification and Characterization* in the revised manuscript to more accurately reflect the content of this section.

- Group Nomenclature: Clarify the rationale for labeling cohorts as Application Group and Discovery Group. Justify these terms in the context of the study design.

🡪 We thank the reviewer for this helpful comment. To improve clarity and align with widely accepted terminology in genetic epidemiology and polygenic risk score research, we have revised the group nomenclature. Specifically, the “Discovery Group” has been renamed as the “Training Set,” and the “Application Group” has been renamed as the “Validation Set.”

In our study design, the **Training Set** comprises individuals without a recorded age at death and was used to identify genetic associations and construct polygenic scores (PRS) for long-term conditions (LTCs). The **Validation Set** consists of an independent subset of participants with recorded age at death, to whom the PRSs were applied for downstream analyses, including associations with lifespan and parental lifespan. This terminology more accurately reflects the roles of each cohort in model development and validation.

This clarification has been added to the Methods section as follows:

“Following these selections, the dataset was divided into two sets: 343,868 individuals without a recorded age at death, designated as the training set, and 34,339 individuals with a recorded age at death, designated as the validation set. The training set was used to identify genetic associations and construct polygenic scores for long-term conditions (LTCs), whereas the validation set was used to apply and validate these scores in relation to lifespan.” (Page 9, lines 13–18)

3. Discussion
Lack of Integration Between Key Findings
The discussion currently presents each result in isolation without synthesizing them into a cohesive narrative. For instance, the GWAS identified HLA as the most significant locus (linked to immunity/inflammation), while the local genetic correlation analysis highlighted LPA as the top candidate. However, the author fails to explore potential biological or mechanistic connections between these findings. A more impactful discussion would:
- Hypothesize how inflammation-related pathways (via HLA) might interact with LPA-mediated lipid metabolism to influence the observed phenotype.

🡪 We thank the reviewer for this helpful suggestion. We have now included a discussion on the potential interaction between inflammation-related and lipid metabolism pathways. Specifically, we note that HLA variants may influence immune activation and chronic inflammation, while LPA variants regulate lipoprotein(a) levels, a pro-atherogenic and pro-inflammatory factor. These findings suggest a potential link between immune and lipid pathways that may contribute to the accumulation of long-term conditions and aging-related outcomes (page 8, lines 16 to 20).

- Address whether these loci operate independently or synergistically in disease pathogenesis, citing prior evidence (e.g., shared pathways like endothelial dysfunction or oxidative stress).

🡪 We have revised the Discussion to address this point. Citing previous studies, we note that HLA and LPA may act through distinct biological mechanisms but could also converge via shared pathways, such as endothelial dysfunction and oxidative stress. While our study design does not directly test their interaction, this evidence supports the possibility of synergistic effects between inflammatory and lipid processes in disease pathogenesis (page 8, lines 13 to 16).

- Propose a research gap: "Given the dual prominence of immune and lipid-related genes, future studies should investigate crosstalk between these pathways in [disease context]."

🡪 We agree with the reviewer’s comment and have added a statement in the *Discussion* highlighting this research gap. Specifically, we note that future studies should investigate whether HLA and LPA loci act independently or synergistically in influencing disease progression, LTC burden, and lifespan. This addition provides a clear direction for future research on the potential immune–lipid crosstalk underlying aging-related multimorbidity (page 8, lines 18 to 22).

Reviewer #4: Dear authors,
The manuscript sheds light on the polygenetic causes that may be related to reduced lifespan in the white ancestry population in the UK by analysis of data from the UK Biobank.
It's noteworthy the large number of data included in the study (343868 alive individuals and 34339 dead individuals).
Some clarity is needed in the following points:
-The term (the long-term condition) is not defined; it includes the risk of diseases that reduce lifespan, the environment and socioeconomic conditions, and the quality of life during the last years before death.

🡪 Thank you for pointing this out. We have now explicitly defined LTCs in the Introduction as chronic diseases or disorders that persist over time, frequently progress, and typically require long-term management. Examples include cardiovascular disease, diabetes, respiratory disorders, and psychiatric conditions. We also clarified that LTC burden refers to the cumulative number of LTCs each individual has in the introduction section as follows: “A number of long-term conditions (LTCs), which are chronic conditions requiring lifelong or long-term management, affect human life expectancy.” (Page 3, lines 2 to 3)

- Why the study depends on the genetic correlation with parents’ lifespan rather than the data of the same individuals in the study.

🡪 We thank the reviewer for this important comment. We used parental lifespan rather than the lifespan of the same individuals to minimize potential bias arising from overlapping sample populations between the exposure (LTC burden) and outcome (lifespan) datasets.

- Please specify the studied genetic loci in a table with their importance or correlation with studied LTC.

🡪 We thank the reviewer for this suggestion. While the primary focus of our study is to examine the overall genetic burden and shared architecture of long-term conditions (LTCs), rather than to dissect individual loci in detail, we have already provided information on variant-level associations in **Supplementary Table 2**, which lists all significant loci and their corresponding effect estimates on the LTC burden.

- The categorization of the 32 LTC in the lines (13-31) is not clear.

🡪 We thank the reviewer for pointing this out. We have now clarified the categorization process in the revised manuscript. Specifically, the 32 long-term conditions (LTCs) were defined using ICD-10 diagnostic codes. This clarification has been added to the Methods section to improve transparency and reproducibility (From page 10, line 23 to page 11, line 21).

- The results of the studied data do not explain if sex has any correlation with lifespan.

🡪 We thank the reviewer for this insightful comment. We have now clarified the relationship between sex and lifespan in the *Results* section. Specifically, we observed a small but statistically significant difference in lifespan between males and females in the application group, with males showing a slightly shorter average age at death (mean 71.0 years, SD = 7.63) compared with females (mean 71.3 years, SD = 7.33; p = 5.0E-04). This finding is consistent with previous evidence suggesting that females tend to have a modest survival advantage. The revised text in the *Results* section now reads as follows:

“Similarly, the average number of LTCs was 1.41 (SD 1.77) in the discovery and 3.54 (SD 2.44) in the application group. The average age at death in the application group was 71.2 years (SD 7.5), with males averaging 71.0 years (SD = 7.63) and females 71.3 years (SD = 7.33). The difference between sexes was statistically significant (p-value = 5.0E-04).” (Page 5, lines 13 to 18)

- The study does not specify which type/s or gene/s of HLA and LPA are the most frequently associated with aging or reduced lifespan.

🡪 We appreciate the reviewer’s valuable comment. We have now clarified the specific HLA variant and LPA gene region that are most strongly associated with the LTC burden or lifespan in the revised manuscript (page 5, lines 20 to 24 and page 7, lines 5 to 7). The corresponding variant annotations, including gene mapping and functional consequence, have now been provided in **Supplementary Table 3**.
