# Supplementary material for: Genetic insights into number of long-term conditions and their relationship with lifespan
Source: PLoS One. 2026 Jan 30;21(1):e0340181. doi: 10.1371/journal.pone.0340181 (PMC12857943; doi:10.1371/journal.pone.0340181)
Supplement: Attachment — Submitted filename: Response to reviewr_2025Dec02.docx [file pone.0340181.s008.docx]

**ACADEMIC EDITOR:**

Dear Academic Editor,

Thank you very much for your time and for coordinating the review process. We appreciate the constructive feedback provided by the reviewers and have revised the manuscript accordingly.

1. Authors need to follow the journal formatting style and guideline, for example, methods before results and discussion.

🡪 We have reorganized the manuscript to comply with the journal formatting guidelines, placing the Methods section before the Results and Discussion. During this revision, we also corrected minor typographical errors and improved clarity where needed.

2. The authors should provide a complete Methods section that can allow the work to be fully reproducible. Additional details may be included in a supplementary section if necessary.

🡪 We carefully revisited the Methods section in response to the comment, and we believe that the current level of detail should allow readers to fully reproduce the analysis.

3. The authors should convert numbers from scientific notation using the “E” format to standard mathematical notation (e.g., 1.77E-176 to 1.77 × 10⁻¹⁷⁶).

🡪 Numerical values previously presented in scientific “E” notation have been converted to standard mathematical notation throughout the manuscript including figure. The only exception is the reporting in the Methods section, where we retained the original format to reflect the tool’s option.
