# Supplementary material for: Genetic insights into number of long-term conditions and their relationship with lifespan
Source: PLoS One. 2026 Jan 30;21(1):e0340181. doi: 10.1371/journal.pone.0340181 (PMC12857943; doi:10.1371/journal.pone.0340181)
Supplement: Attachment — Submitted filename: Response to reviewer_2025Dec08.docx [file pone.0340181.s009.docx]

**ACADEMIC EDITOR:**

Thank you very much for your careful review and for highlighting the remaining issues regarding methodological transparency. We appreciate the need for full reproducibility, and we have now revised the Methods section again to include all essential details that were previously missing. We are grateful for your guidance, and we hope that the updated version meets the journal’s standards.

While the authors have addressed some of my previous comments, the response regarding the Methods section remains insufficient to meet the journal’s standards for full reproducibility, particularly for statistical genetic analyses. Several essential methodological details are still missing. For publication, complete transparency is required.

Below, I outline key areas where further clarification and detail are needed (not exhaustive but provided as guidance).

1. LD Score Regression (LDSC)

The current description of LDSC is not adequate for replication. Please expand this section to include the following details:

- LD score reference panel used (e.g., 1000 Genomes Phase 3 European, UK Biobank-specific LD scores, or custom LD scores).
- Source of LD scores, including links or references.
- SNP filtering criteria (default LDSC filters vs customised filters).
- Treatment of regression intercept, including interpretation of the intercept and how potential sample overlap was assessed or mitigated.
- Any deviations from LDSC default settings, including:
  - handling of high-LD regions (e.g., whether the MHC was removed),
  - use of constrained vs unconstrained intercept,
  - whether genomic control was applied before LDSC.

These parameters meaningfully influence heritability and genetic correlation estimates, and therefore must be clearly reported.

- Thank you very much for the constructive feedback. We have substantially expanded the LDSC section in the revised manuscript, providing full details on the reference panel, LD score sources, filtering criteria, treatment and interpretation of the regression intercept, and any deviations from default settings. We also clarified whether default or customised options were used in each step. For potential sample overlap, we used HDL, which accounts for overlap internally.

2. Local Genetic Correlation Analysis (LAVA)

The description of the LAVA analyses remains minimal. To ensure transparency and reproducibility, please provide:

- The genomic partitioning scheme used:
  - total number of regions,
  - window size,
  - whether the semi-independent LD blocks from Werme et al. (2022) were used or modified.
- LD reference panel used in LAVA (e.g., 1000 Genomes EUR).
- Criteria for region inclusion or exclusion, especially for blocks with low SNP density or unstable LD structure.
- Multiple testing correction approach for determining significant local genetic correlations.
- The exact version of LAVA, including any non-default settings, arguments, or flags.

Local genetic correlation results are highly sensitive to block definitions and LD reference data; thus, full disclosure is required.

- Thank you very much for the helpful comments. In the revised manuscript, we have expanded the description of the LAVA analyses to provide clearer methodological transparency. We now specify the genomic partitioning scheme used and its source, except for window sizes as it vary by block and therefore are not uniform which are provided from LAVA. We additionally describe the LD reference panel, all custom options and steps applied during the analysis and clarify where default settings were used. Details on the multiple testing correction approach, as well as the exact LAVA version and parameters, have also been added.

3. Need for comprehensive methodological transparency across the manuscript

Several additional components of the analysis still lack the detail necessary for reproducibility. The Methods section should allow another researcher to fully reproduce the study without further correspondence. Please ensure that the manuscript reports:

- Full REGENIE parameters for Step 1 and Step 2.
- Complete PRSice-2 settings, including all clumping thresholds, p-value thresholds, and LD parameters.
- Ancestry filtering steps, including thresholds and QC criteria.
- Software environment, including operating system, R/Python versions, and all package versions.
- Where possible, full analysis code or command lines provided via Supplementary Materials or a public repository (e.g., GitHub). This is not mandatory for PLOS ONE when standard methods are used, but adequate procedural detail is required.
- Thank you very much for the constructive feedback. We have expanded the Methods section to provide clearer methodological transparency across all analyses. The revised manuscript now includes detailed descriptions of the parameters used for each tool, specifying custom settings where applied and noting default options when no customisation was used. We have clarified the ancestry filtering procedure, including reliance on UK Biobank–provided ancestry information, and added the R version used in the analyses. Additional details on software and parameter settings have been incorporated to ensure the workflow is fully reproducible.

4. The manuscript would benefit from a more substantive and reflective discussion of its limitations. The current treatment does not adequately address several important constraints inherent in the study design and analytic methods. For example, the reliance on hospital-record-derived phenotypes introduces risks of misclassification, incomplete case capture, and censoring, all of which may affect the accuracy of LTC burden estimates. These issues warrant clearer acknowledgement. Similarly, authors need to mention limitations related to ancestry. Further, the genetic correlation analyses also require more careful framing, as LDSC and LAVA estimate shared genetic signal rather than causality and are sensitive to LD reference panels, block definitions, and SNP density. These interpretive boundaries should be recognised. In addition, the manuscript does not discuss limitations related to genotype QC, imputation quality, or the modest predictive value typically associated with PRS for complex traits. Finally, the discussion presents potential biological implications without balancing them against the environmental and sociodemographic factors that also contribute to LTC burden.

- Thank you sincerely for these thoughtful comments. We have revised the final paragraph of the Discussion to incorporate all of the limitations you highlighted. The updated text now more clearly acknowledges potential phenotype misclassification and incomplete capture associated with hospital-record–based LTC definitions, as well as ancestry-related limitations. We have also added more careful framing of the genetic correlation findings, emphasising that these approaches estimate shared genetic signal rather than causality and are sensitive to LD reference panels, block definitions, and SNP density. In addition, we now discuss limitations related to genotype QC, imputation quality, and the typically modest predictive performance of PRS for complex traits, and we balance the biological interpretation with consideration of relevant environmental and sociodemographic contributors to LTC burden.
